# Gender in Medical Education in Turkey: The Intern Perspective 

**DOI:** 10.30476/jamp.2020.85784.1197

**Published:** 2020-10

**Authors:** OZLEM MIDIK, AYŞEN MELEK AYTUĞ KOŞAN, OZLEM COSKUN, ZEYNEP BAYKAN, ÖZLEM SÜREL KARABILGIN ÖZTÜRKÇÜ, YEŞIM ŞENOL

**Affiliations:** 1 Ondokuz Mayıs University, Faculty of Medicine, Medical Education Department, Samsun, Turkey; 2 Onsekiz Mart University, Faculty of Medicine Medical Education Department, Çanakkale, Turkey; 3 Gazi University, Faculty of Medicine, Medical Education Department, Ankara, Turkey; 4 Erciyes University, Faculty of Medicine, Medical Education Department, Kayseri, Turkey; 5 Ege University, Faculty of Medicine, Medical Education Department, İzmir, Turkey; 6 Akdeniz University, Faculty of Medicine, Medical Education Department, Antalya, Turkey

**Keywords:** Medical student, Curriculum, Gender

## Abstract

**Introdution:**

Gender insensitivity (lack of gender awareness) in the physician’s professional role and practice can lead to outcomes such as gender discrimination and gender-based harassment in various areas, such as medical education, career opportunities, and specialty selection. The purpose of this study was to reveal the place that the concept of gender occupies in medical education in Turkey by canvassing the opinions of final-year medical students regarding theories of gender roles and socialization, academic capitalism, and liberal feminism.

**Methods:**

This study was a Cross-sectional survey. The study population consisted of 1739 interns in six medical faculties in four different geographical regions of Turkey. The reason behind the selection is having different socio-economic factors. Students were selected by simple random sampling technique. For determining it is jumped five students from the lists in faculties. For the validity and reliability of the 14 survey questions, 5 expert opinions were examined and the preliminary instrument was applied to 10 students. Chi-square test was used for comparative analysis.

**Results:**

The students who stated that their gender had not affected their educational lives during clinical training reported that it had adversely impacted their internships (p<0.001). More male students than female ones stated that male physicians were more confidence-inspiring and more industrious, that they managed better, and that they were more likely to recommend a male surgeon. A high number of students reported being undecided on the subjects of concepts or behaviors concerning gender/gender inequality.

**Conclusion:**

We recommend greater focus on role modeling and purposeful teaching of *gender concepts* from the earliest stages of medical education, with particular concentration on gender culture within a process of change involving all hospital personnel in order to prevent gender discrimination.

## Introduction

Discrimination based on gender is the situation where individuals are treated negatively because of their gender and cannot benefit from some opportunities, resources and rights. In a worldwide content throughout their lives, women are subjected to more unequal treatment due to their gender than men. It is observed that women and men who do the same job are also disadvantaged.

Gender roles reflect gendered stereotypes or gender differences determined by society ( 1 - 3 ). This conception is related to cultural views, belief systems, images, and expectations concerning masculinity and femininity imposed by society. Women are expected to concern themselves with children and housework, to be devoted to their husband in a quiet, altruistic, patient, understanding, and affectionate manner, while men are expected to provide for their families, do work requiring physical strength, and to be stern, brave, and logical ( 1 ). Traditionally, females encounter similar expectations in the professional sphere. Teaching, secretarial work, and nursing are regarded as suitable for women, while politics, leadership, and management are professions generally closed to them ( 1 , 3 , 4 ). 

Physicians also behave in accordance with gender roles in their professional lives. For example, female patients are asked about their family more than male patients. This derives from physicians’ perception that family problems are of greater concern to women. Bias affects diagnostic processes, too.

There is a masculine attitude that does not see coronary artery disease as a risk for the woman. While depression is a preliminary diagnosis for women, it is referred to as burnout syndrome for men. Research indicates that physicians are more likely to interpret men’s symptoms as organic and women’s as psychosocial and female patients are assigned more diagnoses of nonspecific symptoms and signs. Women are also prescribed more psychoactive drugs. Physicians exhibit gender-based behaviors not only in relations with patients but also as role models for colleagues, personnel, and students. Gender-aware physicians consider power representations and gender-based expectations and prejudices in such interactions ( 5 ).

The ability of physicians to maintain professionalism depends on their health advocacy and development of personal competence. The school and instructors play a role in the development of professional identity. One particularly important subject in professional identity development is gender awareness. This awareness is essential first for one’s own experience and subsequently in the interests of patients and society. 

Research has shown that gender insensitivity in the physician’s professional role and practice (i.e., a lack of gender awareness) can lead to outcomes such as gender discrimination and gender-based harassment in areas such as medical education, career opportunities, and specialty selection. For example, men constitute the majority of practitioners in surgical fields, while specialties in which women concentrate are traditionally regarded as less prestigious ( 5 - 8 ). 

Gender-related issues have important implications for medical students’ learning. As the importance of gender awareness in medical education has increased, many medical schools have included education concerning gender in their curricula. Various theories investigate the relation between gender and education, and these appear as different research questions ( 2 , 4 ). 

The purpose of this study was to reveal the place that the concept of gender occupies in medical education in Turkey by canvassing the opinions of final-year medical students regarding theories of gender roles and socialization, academic capitalism, and liberal feminism ( 3 ). 

Our research questions within the framework of these theories were as follows: 

RQ 1: What are medical students’ gender-based perceptions during the education process? 

RQ 2: To what extent do members of teaching staff, health personnel and managers, and peers affect gender-based inequalities in medical education? 

RQ 3: Which specialties do medical students regard as appropriate for which gender, and why?

RQ 4: What are medical students’ gender-related perspectives concerning the academic sphere? 

RQ 5: How do medical students regard gender differences in the official and hidden curricula (access, success, preference, deprivation, etc.)? Do medical students think there is any gender discrimination in the program content, method of application, or structuring of exams and tests?

## Methods

A cross-sectional survey was conducted in four different geographical regions; the data was collected between September and November 2018. 

### Study population

This descriptive study was conducted in six medical faculties in four different geographical regions of Turkey. Regional representation was taken into consideration during the selection of medical faculties. The faculty sampling selection was determined by considering cultural and lifestyle options according to the regions in our country. It was chosen from four different regions. The students in the faculty were selected by calculating the 0.90 error margin and 80 power.

The study population consisted of 1739 interns (known as final-year students in Turkey) in these faculties. A sample size of 315 was determined for the identification of difference at an alpha significance level of 0.05, 0.95 power, and 0.5 standard deviation. Internships in final year were taken as clusters and one of the clusters was chosen randomly, and a questionnaire was applied to them. The study population, sample, and collected data are shown in Table 1. 

**Table 1:** Population, sample and collected data numbers

Faculty Name	Population	Sample	Collected data
Ondokuz Mayis University Medical Faculty	251	46	46
Selçuk University Medical Faculty	166	30	35
Erciyes University Medical Faculty	300	54	59
Akdeniz University Medical Faculty	250	45	62
Ege University Medical Faculty	331	60	221
Gazi Medical Faculty	441	80	104
Total	1739	315	527

### Development of the questionnaire

The questionnaire was structured with the participation of all researchers. A conceptual framework was created, and research questions were structured in light of the above-mentioned theories. An item pool was generated and consensus regarding the items of the instrument was established. The questionnaire contained 14 questions and propositions concerning the five research questions based on the theories of gender roles and socialization, academic capitalism, and liberal capitalism. Nine questions were about the socio-demographic features of the participants. One was about the exposure to sexist expressions and behavior during medical education and the other four questions contained propositions concerning the five research questions based on the theories. Scope validity was checked by sending the questionnaire to 5 experts in the survey area prepared.

The preliminary instrument was given to a group of volunteer students (n=10) who evaluated the items in terms of meaningfulness, readability, comprehensibility, sentence length, and clarity of expression. 

The collected data were entered into a standard database by researchers in the relevant faculties. Data quality control was performed, and the final data were collected and analyzed.

### Data analysis

Statistical analysis was performed on PASW Statistics software for Windows (SPSS, Inc., IBM), version 18.0. Mean values and standard deviation were calculated for continuous variables, while descriptive analyses (number and percentage) were given for nominal variables. The chi square test was used for comparative analysis.

**Ethical Considerations:** This study was approved by the Ethics Committee of the X University Faculty of Medicine (Decision Number 11-6.1/10). There were no conflicts of interest in this study.

## Results

The students’ socio-demographic characteristics are presented in Table 1. 

We found that 60.5% of female students and 39.5% of male students thought that insufficient importance was attached to the concept of gender equality during medical education, (2 = 11.603, p = 0.003). In response to the question “How has your gender affected your working and educational life during clinical training?”, no difference was found between female and male students (2 = 3.439, p = 0.179). In addition, 54.3% (n = 286) of interns reported that their gender had no effect on their working and educational lives during their internship, 26.2% (n = 138) said it had an adverse impact, and 18.6% (n = 98) reported a positive effect. Additionally, 47.6% of students who stated that their gender had no effect on their educational lives during clinical training reported a negative impact during their internship. The change between the clinical and internship periods was statistically significant (2 = 111.208, p < 0.0001). The proportion of female students believing that their gender had a negative impact on their working and educational life during their internship (71.7%) was higher than that of male students (28.3%) (2 = 40.997, p < 0.001).

A significant difference between gendered behaviors was found only in the context of health personnel (=214,884, p<0.001), with 40.1% of female students and 27.7% of male students reporting experiencing gendered behavior by health personnel (Table 2).

**Table 2:** Exposure to sexist expressions and behavior during interns’ medical education

	Yes	No	undecided
	N (%)	N (%)	N (%)
I have encountered gendered discourse and behaviors by my peers during my medical education (n=526).	216 (41.1)	253 (48.1)	57 (10.8)
I have encountered gendered discourse and behaviors by members of teaching staff during my medical education (n=527).	215 (40.8)	233 (44.2)	79 (15.0)
I have encountered gendered discourse and behaviors by health personnel during my medical education (n=527).	179 (34.0)	259 (49.1)	89 (16.9)
I have encountered gendered discourse in various situations (presentations, discussions, at the patient bedside, etc.) during my medical education (n=526).	132 (25.1)	322 (61.2)	72 (13.7)
I have encountered gendered discourse and behavior by deans and assistant deans during my medical education (n=527).	38 (7.2)	417 (79.1)	72 (13.7)

Thirty percent (158) of students stated that a female physician graduating from a medical school can select whatever specialty she wishes. The great majority of students (410 students; 77.79%) described internal medical sciences as the most suitable specialty for a female physician (Table 3).

**Table 3:** Statements regarding fields suitable for female physicians

Conceptions	Supporting quotation
Physical feature	For example, fields such as cardiovascular surgery and plastic surgery require a male body.
	Women already predominate in dermatology and pediatric diseases due to their lower rates of mental and physiological burnout. So they should not enter this field, too.
	They will be mentally and physically worn out, as part of their nature, by shifts and a heavy workload.
Family	Basic sciences are more suited to family life. It is easier to cope with children.
	Women are not popular in surgical branches, because they become pregnant and drop out.
Skills	Due to the small number of invasive procedures.
	Women have better fine motor skills.
Character features such as affection, concern and communication	They find it easier to establish communication with children because of their maternal affection.
	They are emotional.
	They are more suited to emotional women who cannot tolerate stress.
	They have powerful productivity.
Violence	They are less exposed to physical violence.
	Their shifts are easy and they have a lower likelihood of experiencing violence.
Beauty	Women are suited to plastic surgery because they have a better understanding of arrangement and beauty.
	I think that women are better, particularly in cosmetic areas.
Customs, culture, perception, prejudice	Conservative trend
	A desire for female doctors in a less developed society, such as Turkey.
	A heteronormative male hierarchy due to the inconsistent behavior of male physicians toward female physicians in surgical branches – because I have never seen a female urologist.

Our results showed that 48.6% (n=256) of students had considered selecting a specialty when starting medical school. The departments they most considered selecting, in a descending order of preference, were pediatrics, ophthalmology, and gynecology. However, upon completing medical school, 10.2% of students (n=54) reported not considering specializing. The most popular departments, in a descending order of preference, among those students considering specializing were ophthalmology, pediatrics, and internal diseases. On entering the medical faculty, 46.3% of female students and 61.0% of male students desired to enter a surgical branch (2=4.978, p=0.026), while on completing the faculty 42.6% of female students and 55.4% of male students continued to prefer a surgical branch (2=6.828, p=0.009) (Table 4).

**Table 4:** Students’ specialization preferences

Science branches	Entering the faculty n=525	Finishing the faculty n=521
	N (%)	N (%)
Basic	3 (0.6)	11 (2.1)
Internal	112 (21.3)	214 (40.6)
Surgical	124 (23.5)	202 (38.3)
Undecided	17 (3.2)	37 (7.0)
Total	256 (48.6)	464 (88.0)

Although more than half of the students (mean 76.6%) stated that gender was of no importance in the roles described, more roles were attributed to men in branches with intense involvement of surgical sciences and invasive procedures. Nursing, counseling, and basic sciences were regarded as more suitable for women (Table 5).

**Table 5:** The genders that interns prefer for physician roles

	Female	Male	Intersex	Gender is of no importance
	n(%)	n(%)	n(%)	n(%)
Medical science (n=523)	23 (4.4)	44 (8.4)	9 (1.7)	447 (85.5)
Counseling (n=524)	65 (12.4)	38 (7.2)	7 (1.3)	414 (79.1)
Role model (n=524)	54 (10.3)	62 (11.8)	8 (1.5)	400 (76.4)
Branches involving intensive invasive procedures (n=524)	16 (3.0)	163 (31.1)	7 (1.3)	338 (64.6)
Academia (n=523)	29 (5.5)	26 (5.0)	10 (1.9)	458 (87.6)
Basic sciences (n=522)	68 (13.0)	23 (4.4)	8 (1.5)	423 (81.1)
Surgical sciences (general surgery, orthopedics, urology, etc.) (n=524)	5 (0.9)	222 (42.4)	3 (0.6)	294 (56.1)
Internal sciences (n=524)	36 (6.8)	24 (4.6)	11 (2.1)	453 (86.5)
Nursing (n=522)	142 (27.2)	17 (3.2)	8 (1.5)	355 (68.1)
A team colleague I have worked or kept notes with (n=524)	58 (11.1)	55 (10.5	7 (1.3)	404 (77.2)
An assistant physician I have worked or kept notes with (n=523)	37 (7.1)	58 (11.1)	6 (1.1)	423 (80.7)

The interns’ perceptions concerning physicians also varied by gender. More female students than male ones thought that women were more subjected to harassment (2=6.377, p=0.012), and that women were more exposed to occupational violence because of their gender (2=11.506, p=0.003). In addition, 96.3% of women and 72.1% of men thought that women were under greater stress due to workload and family responsibilities (2=31.925, p<0.001); students also stated that female physicians established greater emotional bonds with patients (2=17,469, p<0.001) (Table 6).

**Table 6:** Interns’ gender-based perceptions regarding physicians and medicine

Female	Male	Both female and male	
n(%)	n(%)	n(%)	
105 (20.2)	7 (1.3)	408 (78.5)	….physicians are subjected to mobbing (n=520)
60 (11.4)	25 (4.8)	439 (83.8)	….physicians are polite toward patients (n=524)
234 (44.9)	17 (3.3)	270 (51.8)	….physicians establish greater emotional bonds with patients (n=521)
229 (43.8)	35 (6.7)	259 (49.5)	….physicians are more stressed due to workload and family responsibility (n=523)
18 (3.4)	71 (13.6)	434 (83.0)	....physicians inspire greater confidence (n=523)
5 (1.0)	124 (23.8)	391(75.2)	....physicians earn more money (n=520)
5 (0.9)	126 (23.9)	392(74.4)	....physicians are more effective in emergency situations (n=523)
24 (4.6)	114 (21.6)	385 (73.1)	....physicians make good managers (n=523)
40 (7.6)	58 (11)	422 (80.1)	….physicians are harder working (n=520)
249 (47.2)	25 (4.7)	246 (46.7)	....take greater care over dress and appearance because of professional anxieties (n=520)
186 (35.3)	84 (15.9)	252 (47.8)	....are more subjected to occupational violence because of their gender (n=522)
338 (64.1)	16 (3.0)	165 (31.3)	....physicians are more subjected to disturbing behavior from the opposite sex in their professional lives due to their gender (n=519)
186 (35.3)	41 (7.8)	292 (55.4)	….physicians cause greater workforce losses because of their biological and social characteristics (such as military service and giving birth) (n=519)
32 (6.1)	122 (23.1)	365 (69.3)	….physicians are more respected and appreciated by management (n=519)
58 (11.0)	35 (6.6)	415 (78.7)	Individuals are more successful when their spouses are doctors (n=508)
205 (38.9)	9 (1.7)	305 (57.9)	….physicians’ professions play a role in their decisions to have children (n=519)
39 (7.4)	93 (17.6)	377 (71.5)	…physicians support their male colleagues more in professional matters (n=509)
248 (47.1)	18 (3.4)	251 (47.6)	…physicians work in easier fields with fewer shifts (517)
25 (4.7)	149 (28.3)	344 (65.3)	Medicine is a branch of science dominated by ……physicians (n=518)
22 (4.2)	172 (32.6)	317 (60.2)	physicians can live without occupational anxieties because of their gender (n=511)
18 (3.4)	89 (16.9)	414 (78.6)	I would recommend a …… physician to my patients/colleagues (n=521)

However, more male students than female ones thought that male physicians inspired greater confidence (2=13.549, p=0.001), worked harder (2=35.817, p<0.001), were good managers (2=23.332, p<0.001), and could give patients or relatives more advice than a female surgeon (2=20.218, p<0.001). 

More female students than male ones thought that women academics encountered more gendered behavior (2=14.954, p<0.001) and that female academics had greater responsibility for student education (2=8.684, p=0.013) (Table 7).

**Table 7:** Interns’ perceptions toward academics

Female	Male	Both female and male	
n(%)	n(%)	n(%)	
60 (11.4)	20 (3.8)	438 (83.1)	…academics establish better communication with patients (n=518)
105 (19.9)	28 (5.3)	388 (73.6)	…academics are more affectionate toward their patients (n=521)
57 (10.8)	46 (8.7)	417 (79.1)	…academics establish better relations with colleagues (n=520)
46 (8.7)	44 (8.3)	430 (81.6)	…academics establish better communication with students (n=520)
44 (8.3)	53 (10.1)	422 (80.1)	…academics are more inspirational as role models (n=519)
50 (9.5)	51 (9.7)	415 (78.7)	…academics assume greater responsibility for students’ education (n=516)
60 (11.4)	36 (6.8)	406 (77)	…academics take education more seriously than male academics (n=516)
39 (7.4)	50 (9.5)	425 (80.6)	…academics are better educators (n=514)
21 (4.0)	93( 17.6)	400 (75.9)	…academics have greater leadership qualities (n=514)
14 (2.7)	51 (9.7)	441 (83.7)	…academics are better leaders than women in their fields (in terms of specialties) (n=509)
42 (8.0)	32 (6.1)	443 (84.1)	...academics give better counseling (n=517)
28 (5.3)	46 (8.7)	444 (84.3)	…academics conduct better projects (n=518)
11 (2.1)	74 (14)	432 (82.0)	…academics have economic privileges (n=517)
11 (2.1)	94 (17.8)	410 (77.8)	…academics have political privileges (n=515)
10 (1.9)	87 (16.5)	421 (79.9)	…academics have better relations with technology (n=518)
12 (2.3)	52 (9.9)	450 (85.4)	…academics make names for themselves due to making discoveries (n=514)
19 (3.6)	75 (14.2)	423 (80.3)	…academics have more initiative (n=517)
121 (23)	24 (4.6)	372( 70.6)	….academics are better in the social aspects of medicine (such as public health and medical education) (n=517)
88 (16.7)	53 (10.1)	377 (71.5)	…academics are more competitive (n=518)
101 (19.2)	43 (8.2)	375 (71.2)	… academics are keen (n=519)
264 (50.1)	18 (3.4)	233 ( 44.2)	…academics experience more sexist behavior and attitudes (n=515)
22 (4.2)	88 (16.7)	404 (76.7)	…academics’ projects are better supported (n=514)
35 (6.6)	50 (90.5)	433 (82.2)	…academics are more stable in terms of academic success (n=518)

More male students than female ones thought that female academics had greater leadership qualities (2=15.151, p<0.001), that male academics were better than women in their fields (in terms of specialty knowledge) (2=7.955, p=0.019), that female academics provided better counseling (2=22.816, p<0.001), and that male academics produced better projects (2=9.059, p=0.011) and took more initiatives (2=14.462, p<0.001). 

Our results showed that 8.6% of female students and 3.8% of male students thought that gender affects medical faculty selection (2=4.995, p=0.025), while 46.9% of female students and 60.9% of male students thought that gender equality prevailed in the medical education they received (2=10.073, p=0.002).

More male students than female ones stated that being male or female affected examination success (2=5.919, p=0.015) and that female medical students used their sexuality to pass classes (2=21.745, p<0.001). 

More female students than male ones reported being told that surgical branches were more suitable for male students (2 = 10.447, p = 0.001), or that “You cannot do that” (2=31.910, p<0.001). They also thought that faculty administrations wished to see more male students in student representative bodies (2=5.887, p=0.015) (Table 8). 

**Table 8:** Interns’ perceptions concerning medical education (n:517)

	I entirely agree	I agree	I am undecided	I disagree	I entirely disagree
My gender had an effect on my selecting medical school.	2.3	4.1	6.4	3.9	83.3
Medical education that I take care of gender equality.	32.6	19.5	24.7	28.	12.9
Work is distributed irrespective of gender during internship.	39.1	13.5	18.6	9.7	16.9
Members of teaching staff or assistant physicians prefer working with male medical students during internship.	8.2	11.6	24.3	15.4	38.1
Female students are more subjected to sex discrimination than male students.	25.4	21.6	21.1	10.2	19.5
Being a man or a woman affects one’s success in class.	9.1	9.1	17.3	10.6	51.8
Female medical students use their sexuality to get on in class.	5.5	9.1	12.5	17.5	53.1
Members of teaching staff are egalitarian in class and do not have sexist attitudes.	19.4	9.9	24.7	24.1	19.2
Members of teaching staff working in surgical branches emphasize that surgical branches are more suited to men.	35.1	25.8	18.4	86.5	12.3
The words ‘You could not do it’ are addressed more to female students than male students.	21.1	17.5	19.4	13.5	25.6
Faculty administrations want to see male students rather than female students on student representative bodies.	7.6	9.1	20.5	14.6	45.9
Female members of teaching staff do not like female students.	8.2	13.3	26.4	20.7	28.8
Students can benefit from extra opportunities in medical educations (such as taking part in congresses) without gender discrimination.	38	21.4	18.6	5.5	14.4
Class materials (presentations, member of staff handouts, videos) contain sexist language.	5.1	10.4	16.3	16.1	50.1
Members of teaching staff are egalitarian in small group education sessions and do not exhibit sexist attitudes.	31.7	20.5	21.1	9.7	14.8

## Discussion

This study considered gender in medical education in Turkey from the intern perspective through eclectic theories of roles and socialization, academic capitalism, and liberal feminism. 

More female interns than males in our study considered that particular importance was not attached to the concept of gender equality. Similarly, numerous studies have reported that experiences of gender bias are more commonly described by women ( 9 - 11 ). Male students have been reported to be less informed about gender and to be more skeptical concerning gendered approaches in medical education. In addition, male students appear to be less motivated to reflect on gender-related values and attitudes ( 12 - 15 ). A similar finding, that male students are less aware of the concept of gender than female students, was also observed in our study. However, this should be interpreted not as indicating male insensitivity but rather as a problem of the medical education process. This is because in addition to their own beliefs during the learning process, medical students also acquire beliefs and behaviors from the school and their teachers, directly or otherwise. These acquisitions within an individual may not always be positive. Developing awareness and change is regarded as the primary means of bringing out from hidden perceptions that may be filled with prejudices or stereotypes. It may therefore be concluded that this awareness makes important contributions to these prejudices. 

Students stating that their gender has an adverse effect on their educational lives also reported that this was more marked during their internship than during the clinical period, and was more frequently reported by female students. Students reporting that gender discrimination was greater during their internship than in the clinical period reveal a difference between structured learning processes and opportunistic ones and indicate the real-world scenario. The difference between the periods is thought to result from interprofessional communication in a chaotic environment. Members of the teaching staff, managers, and peers all appear to have similar levels of impact on gender-based inequalities in medical education in Turkey. Surprisingly, students are exposed to the sexist comments and behavior from health personnel, and the level is again higher among female students. This makes it essential for work on gender inequality in medical education to be considered from a broader perspective. Spreading gender awareness as an institutional culture reveals alertness in all learning and work activities. 

In our study, 77.8% of the students described internal medical sciences as the most suitable specialty for a female physician. The students most commonly regarded dermatology, family medicine, and pediatrics as appropriate branches for women. This may result from the attitude that they regard women incapable of heavy work and delicate creatures who should not fatigue themselves, and that they equate women with the family, children, affection, concern, and goodness. Students regarded obstetrics, one of the surgical medical fields, as the fourth most appropriate field for women. Students who did not regard male-dominated fields such as orthopedics and urology suitable for women placed obstetrics at the head of that list, saying that “women prefer female physicians.” 

This study determined that students regarded orthopedics, one of the male-dominated fields, as unsuitable for female physicians. O’Connor investigated the reason for the very few female students in the field of orthopedics and concluded that “medical school experiences shape women students’ interest in Orthopedic Surgery.” Male students reported taking an interest in the field before their clinical rotations, while female students stated that they were more affected during their rotations. The author also reported that negative perceptions of orthopedics, such as involving a difficult lifestyle and requiring physical strength, together with the male domination of the field, reduced female students’ interest in it. Finally, the author recommended that female students be motivated during rotations by interacting with female role models ( 16 ). 

Although the majority of students reported that gender was unimportant in the defined roles, males were indicated as role models for surgical sciences and branches involving intensive, invasive procedures. Nursing, counseling, and basic sciences were regarded as more suited to women. These findings show that women are similarly equated with motherhood and roles involving care and affection, and that the roles imposed by society persist during medical education. 

The female students stated that woman were more exposed to harassment and experience greater occupational violence, and that female physicians experienced greater stress due to workload and family responsibilities, and also that they established more emotional bonds with patients. 

The male students stated that male physicians inspired greater confidence, worked harder, and managed better, and that male surgeons provided more advice for patients and relatives. These findings show that all interns, male and female, possess stereotypical prejudices regarding physicians and medicine. Female students have greater gender sensitivity than males, but both genders have stereotypic conceptions. Anderson, et al. reported that German students exhibited gender sensitivity, but that they also possessed gender-stereotypical attitudes toward patients and doctors ( 15 ). Swedish female students have been shown to have a lower level of stereotypical ideas. There is also evidence that gender-stereotypical ideas are higher in men than in women ( 13 ).

The students also perceived gendered behavior in a similar manner in academia. More female students than male ones stated that female academics encountered more gender-based behavior and attitudes and thought that female academics were more responsible for education. 

More male students than female ones stated that female academics had greater leadership qualities, that male academics were better than female academics in their fields (in terms of specialty), that female academics provided better counseling, and that male academics were better at carrying out projects and took more initiatives. Awareness of encountering sexist behavior and attitudes was higher among female students. The male students tended to attribute leadership and initiative to men and counseling to women in exhibiting gendered behavior. Nora, et al. reported that gender discrimination and sexual harassment were widespread in academic medical centers, particularly during the clinical period, and that male students perceived these more frequently than women ( 17 ). 

Although it has been alleged that female surgeons tend to prioritize their families over their careers, research has reported that women are just as qualified as men to assume leadership positions and are more desirous to do so than men. However, traditional gender roles, gender discrimination, and a lack of effective mentors prevent these desires from being fulfilled ( 18 ). 

Our results showed that 46.3% of women wished to enter a surgical branch when first entering a medical school, and that 42.6% indeed did so upon graduating. Cynicism in the desire to enter surgical branches is higher among women than among men. This shows that female students lose motivation and abandon their dreams during the learning process for numerous reasons, known and/or unknown.

The surgical branch most popular with women in the literature is obstetrics, followed by ocular diseases. However, approximately 74% of women who selected surgical branches reported that the home-work balance adversely affected their careers; 81.4% reported that working in the workplace together with the home constituted a heavy burden; 38.4% believed that men were more successful in jobs requiring active strength, and 61.2% reported that surgical branches, with their long working hours and shifts, were better suited to men. Society expects men and women to adopt roles that involve behaviors, attitudes, and characteristics associated with their gender. The gender role models imposed by society can lead to occupational sex discrimination in profession selection ( 19 ).

 The current study had several limitations. It was conceptualized as an attempt to gain information regarding the gender of medical students in four different geographical regions. Thus, comparison with a control group of students from other geographical regions was not sought at this point. 

Although male students asserted that gender equality was maintained in the medical education they receive, a larger number reported that being a male or female student affects success in class and that female medical students used their sexuality to pass classes. This reveals that women are regarded as sex objects in professional education and shows a perception that female students use this characteristic in their own favor. 

The female students who were more inclined to think that gender equality was not observed in the medical education they received stated that their gender had an effect on their selection of medical school. The female students thought that members of the teaching staff emphasized that surgical branches were more appropriate for male students, that the words “You could not do that” were addressed more to female students, and that male students were more represented in student bodies. 

Gender bias is encountered in medical textbooks, medical curricula, and in other education tools and materials and has an adverse impact on individual attitudes and decision making processes, which is in turn reflected in students’ career opportunities and expectations ( 9 ). The participants in this study stated that academics encouraged students on the basis of their gender in the selection of specialty fields. 

## Conclusion

One of the important findings of this study was the high number of interns describing themselves as undecided/uncertain. This indicates that they do not think of concepts or behaviors related to gender/gender equality from a critical perspective, that they regard these as generally accepted, and that students have a low level of awareness. 

If the gender discrimination is seen in medical practices, as in life itself, it has to be prevented. It is essential that gender awareness be facilitated from the earliest stages of the medical education process.

Gender awareness must be included in the medical education spiral curriculum, and schools and educators must include more positive models and purposeful teaching during the internship period. More work is needed on gender culture during this change process. 
